# The aqueous extract of aged black garlic ameliorates colistin-induced acute kidney injury in rats

**DOI:** 10.1080/0886022X.2018.1561375

**Published:** 2019-02-04

**Authors:** Tae Won Lee, Eunjin Bae, Jin Hyun Kim, Ha Nee Jang, Hyun Seop Cho, Se-Ho Chang, Dong Jun Park

**Affiliations:** a Department of Internal Medicine, Gyeongsang National University Changwon Hospital, Changwon, Republic of Korea;; b Biomedical Research Institute, Gyeongsang National University Hospital, Jinju, Republic of Korea;; c Institute of Health Sciences, Gyeongsang National University, Jinju, Republic of Korea;; d Department of Internal Medicine, Gyeongsang National University Hospital, Jinju, Republic of Korea;; e Department of Internal Medicine, College of Medicine, Gyeongsang National University, Jinju, Republic of Korea

**Keywords:** Colistin, garlic, inflammation, nephrotoxicity, oxidative stress

## Abstract

The use of colistin in the treatment of multidrug-resistant Gram-negative bacterial infections is restricted due to nephrotoxicity. We investigated the effects of aged black garlic extract (ABGE) on colistin-induced kidney injury in rats. Rats were assigned to four groups. Normal saline was intraperitoneally and intragastrically injected for control group. ABGE was intragastrically injected for garlic group. Ten mg/kg of colistin was intraperitoneally injected for 6 consecutive days for colistin group. One percent of ABGE was done 30 min prior to colistin injection for treatment group. Rats were sacrificed on the next day after last colistin injection. Colistin injection increased the serum levels of blood urea nitrogen and creatinine; however, ABGE prevented deterioration of these serum levels. ABGE also alleviated tubular damage, including vacuolation and necrosis. TUNEL-positive cells were observed less frequently for the ABGE-treated groups. CD68 positive cells were significantly decreased by pretreatment with ABGE. Levels of oxidative stress biomarkers such as 8-hydroxydeoxyguanosine and malondialdehyde were lower in the ABGE-treated groups. Levels of NF-*κ*B, inducible NO synthase, COX-2, and TGF-β1 were lower in rats that had been treated with ABGE injection. Renal levels of IL-1*β* and TNF-*α* were increased by colistin administration whereas renal SOD, catalase, and GSH levels were restored by ABGE administration. These results suggest that ABGE, which has antioxidant and anti-inflammatory properties, might be a potential therapeutic agent to prevent renal toxicity of colistin.

## Introduction

Colistin recently has been an integral antibiotics after the appearance of gram-negative bacteria such as *Pseudomonas aeruginosa*, *Acinetobacter baumannii*, and *Klebsiella pneumoniae* which are resistant to all classes of available antibiotics [1–3]. However, nephrotoxicity is the most frequent and grave side effect resulting in either early discontinuation of administration or worse prognosis [3,4]. The rates of nephrotoxicity were various from 45% to 55% according to currently recommended colistin regimens [5–8] although a recent clinical population pharmacokinetic study revealed these dosage regimens are not sufficient in many patients [9]. Even if the mechanism of colistin-induced nephrotoxicity has not been accurately known, it seems to be correlated with total dose and/or duration of colistin administration [6,10].

Garlic (*Allium sativum*) has been known as a medicinal food because of various biological effects such as antioxidant, anti-inflammatory, antimicrobial, antithrombotic, and antitumor effects [11–16]. Recently, aged black garlic extract (ABGE) which is produced through aging procedures at high temperature and humidity over a long time resulting in containing high levels of organosulfur compounds has also antioxidant and anti-inflammatory effects [17–19]. Under the unique process, raw garlic possessing a strong flavor and pungent odor is turned into sweet and odorless form which is good to eat. Some reno-protective effect of garlic or certain constituents from garlic has been studied [20–25]. However, there have no studies for reno-protective effect of ABGE, especially colistin renal toxicity animal model. Therefore, this study was designed to determine whether ABGE has reno-protective effect against toxicity of colistin to kidney by determining laboratory parameters and performing histological examinations.

## Materials and methods

### Preparation of aged black garlic extract (ABGE)

For BG preparation, fresh garlic bulb was incubated for 48–60 h at 80–90 °C followed by 48–60 h at 70–80 °C, then 72–120 h at 60–70 °C, and finally 72–120 h at 55–65 °C. ABG was suspended with 10 volumes of distilled water. The suspended ABG was extracted for 5 h at 95 ± 3 °C. The water extract was filtered twice through four pieces of Cheesecloth (Kavon Filter Products Co., Farmingdale, NJ), freeze-dried, powdered, and stored at 15 ± 3 °C until further analysis.

### Animals and experimental designs

Animal studies were conducted according to the guidelines for the care and handling of animals prepared by the Gyeongsang National University Institutional Animal Care & ethics committee (GNU-120615-R0024) and followed the NIH publication ‘Principles of Laboratory Animal Care’. Ten-week-old male Sprague Dawley rats were purchased from Koatech Inc. (Peongtaek, South Korea). JH Shin affiliated to Namhae Garlic Research Institute (Namhae, South Korea) kindly donated ABGE. Rats were divided randomly into four different groups; Control group (*n* = 7), normal saline was intraperitoneally injected for 6 consecutive days like colistin injection and also was intragastrically injected prior to 30 min colistin injection for 6 consecutive days like ABGE injection. Garlic group (*n* = 7), 1% of ABGE (100 *µ*L per individual) was intragastrically injected. Colistin group (*n* = 7), 10 mg/kg of colistin (STERI*MAX* INC., Ontarino, Canada) was intraperitoneally injected for 6 consecutive days. Garlic plus colistin group (*n* = 7); ABGE injection was intragastrically done prior to 30 min colistin injection for 6 consecutive days. On the 24 h after last colistin injection, all rats were sacrificed, blood samples were collected for renal function, and the kidney tissues were fixed for histological examination including Masson’s trichrome, TUNEL, and immunohistochemical staining and were frozen for ELISA.

### Protein preparation and measurement of inflammatory cytokines and antioxidants

The previously removed kidneys were extracted by homogenization in lysis buffer [1× PBS (pH 7.4) with 1% Triton X-100 and 1 mM EDTA] containing 10 *µ*M leupeptin and 200 *µ*M PMSF. The lysates were sonicated several times for 3–5 min each and centrifuged at 12,000 rpm for 20 min at 4 °C. The supernatants were collected and the protein concentration of each lysate was determined using a bicinchoninic acid protein assay kit (Pierce, Rockford IL) according to the manufacturer’s protocol. Bovine serum albumin was used as a standard. Levels of IL-1*β* (Elabscience, Houston, TX), TNF-*α* (Elabscience), superoxide dismutase (SOD, Cell Biolabs Inc. San Diego, CA), catalase (Cell Biolabs Inc.), and glutathione (GSH; Cell Biolabs Inc.) in the kidneys were measured by using a specific ELISA kit and performed according to the manufacturer’s instructions.

### Biochemical tests

Renal function was assessed by measuring serum blood urea nitrogen (BUN) and creatinine levels using standard diagnostic kits in an automatic analyzer (ADIVA 1650, Bayer, Japan).

### Histological analyses

Tubular injury was assessed in periodic Masson’s trichrome-stained kidney sections. Tubular injury score was previously described [26]. As briefly, tubular injury was defined as tubular vacuolation and tubular epithelial necrosis. Tubular injury has been scored by grading the percentage of affected tubules under ×400 magnification; 0, 0%; 0.5, <10%; 1, 10–25%; 2, 26–50%; 3, 51–75%; 4, 75–100%. To score injured tubules, whole tubular numbers per field were considered as standard under ×400 magnification. The grading percentage was calculated in each field as follows: injury score (%) = (numbers of injured tubules/number of whole tubules) × 100. At least 10 areas in the cortex per slide were randomly selected.

### Renal apoptosis

The degree of renal apoptosis was examined by TUNEL assay. Detection of DNA fragmentation was performed using a kit (Roche Applied Sciences, Indianapolis, IN). TUNEL-positive cells were identified through the nucleus, which was stained brown. The signals were analyzed by a blinded observer using NIS Elements BR3.2 (Nikon, Japan) software in 10 randomly selected fields.

### Immunohistochemical staining for renal inflammation and oxidative stress

Immunohistochemical detection was conducted on the Avidin-Biotinylated-HRP complex kits (ABC; Vector Laboratories, Burlingame, CA). The sections were incubated with 1% normal serum and then treated successively with each primary antibody; anti-8-hydroxydeoxyguanosine (8-OHdG) (Abcam, Dawinbio Inc., Korea), anti-malondialdehyde (MDA) (Abcam), anti-ED-1 (Santa Cruz Biotechnology, Santa Cruz, CA), anti-transforming growth factor TGF-*β*1 (Abcam), anti-phospho-nuclear factor kappa beta (NF-*κ*B) (Santa Cruz Biotechnology), anti-COX-2 (Cell Signaling Technology, Danvers, MA), and anti-inducible NO synthase (Santa Cruz Biotechnology) at 4 °C for 16 h. After the sections were washed in, they were incubated with secondary antibody at room temperature for 90 min. Finally, the sections were incubated with ABC for 60 min at room temperature, rinsed in PBS, counterstained with hematoxylin, and developed by 3,3-diaminobenzidine tetrahydrochloride with H_2_O_2_. Stained kidney sections were captured using the virtual microscopy system (Nikon eclipse 80i, Tokyo, Japan).

### Statistical analysis

All results are presented as mean ± SE (*n* = 7). Statistical analyses were conducted using the GraphPad Prism (GraphPad Software, La Jolla, CA). Statistical differences between the groups were tested by One-way analysis of variance followed by Tukey’s Multiple Comparison Test. Statistical significance was defined as *p* < 0.05.

## Results

### Renal function

**Figure 1.**
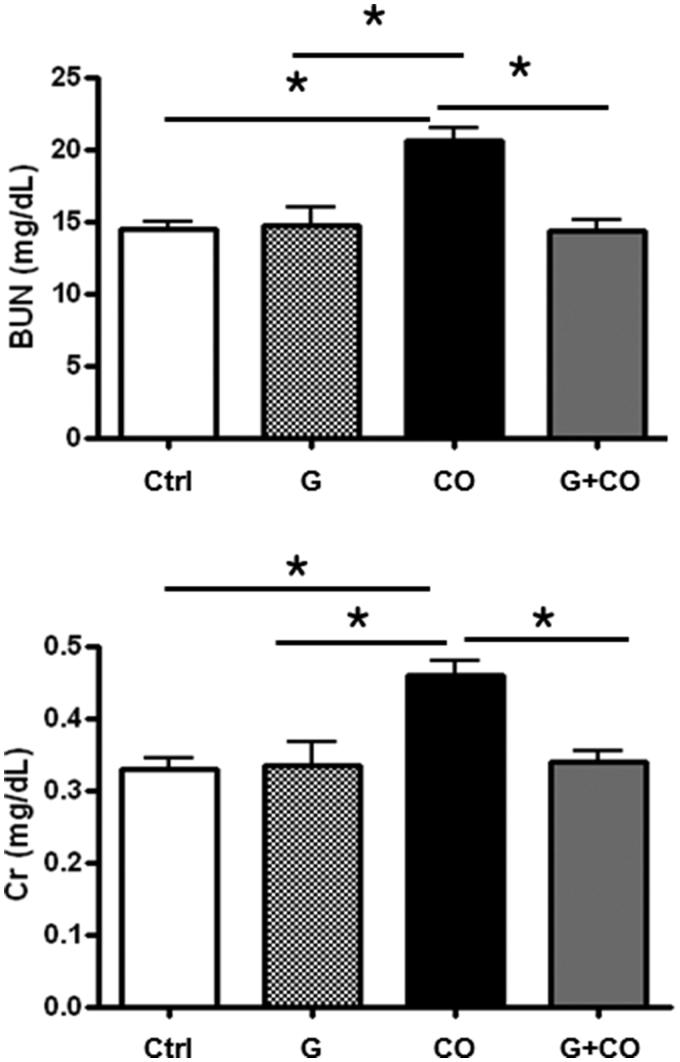
The levels of BUN and Cr in the serum of rats. Blood was collected on the seventh day after colistin administration. Twenty-eight rats were randomly assigned into four different groups: Ctrl: 1 ml/kg intraperitoneal (i.p.) injection of sterile normal saline; G: intragastric administration of saline solution with 1% ABGE (100 *µ*L per individual); CO: 10 mg/kg i.p. injection of colistin; G + CO; intragastric saline solution with 1% ABGE (*µ*L per individual) and 10 mg/kg i.p. injection of colistin for 6 consecutive days. Data are expressed as the mean ± SE (*n* = 7). *p < 0.05.

The administration of colistin led to a rise in BUN from 5.4 mmol/L (baseline) to 7.5 mmol/L, and a rise in serum creatinine (Cr) from 26.5 µmol/L (baseline) to 44.2 µmol/L at 7 days after colistin injection, compared with controls and the ABGE**-**only treatment group. In contrast, garlic-pretreated rats showed significant attenuation in BUN and serum Cr elevation (7.5 mmol/L vs. 5.4 mmol/L, *p* < 0.001 for BUN; 44.2 µmol/L vs. 26.5 µmol/L, *p* < 0.001 for Cr) (Figure 1) at 7 days, compared with colistin alone. The results indicated that garlic pretreatment protected against colistin-induced nephrotoxicity.

### Renal histology

**Figure 2.**
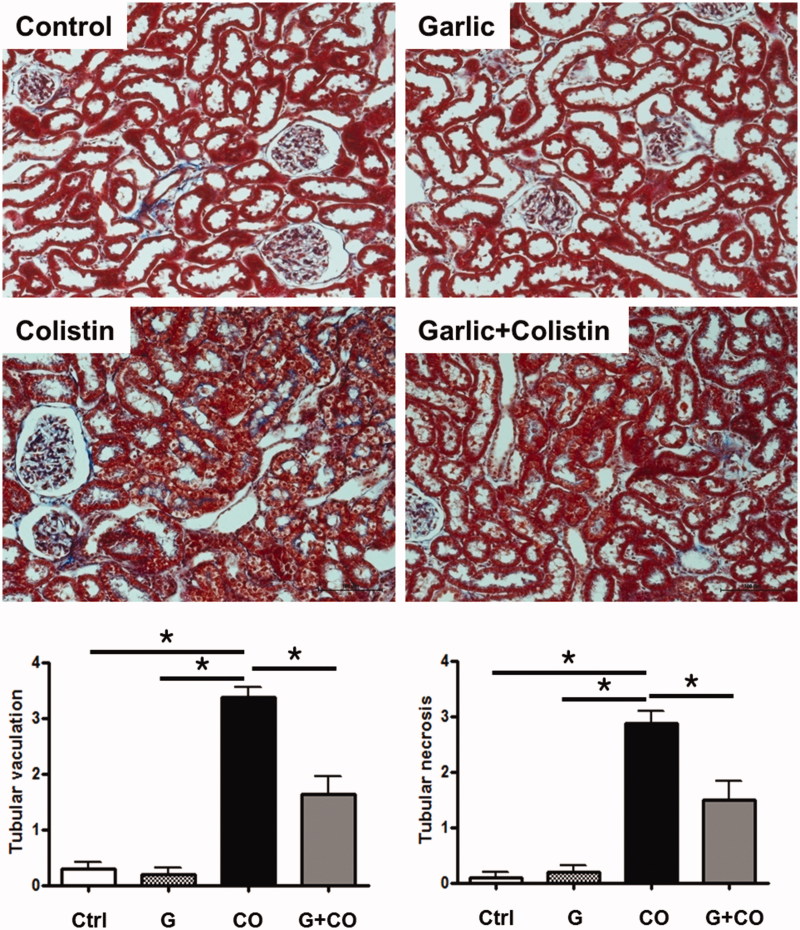
Decreased tubular damages and renal fibrosis by ABGE in the kidney with colistin administration. Masson’s trichrome staining was performed in the sections of rat kidneys 7 days after colistin administration (original magnification × 200). The method of tubular injury scoring and grading were described in the ‘Materials and Methods’ section. Figure shown is representative of four groups. Values are expressed as means ± SE (*p < 0.05). Scale bar, 100 μm.

Rats that received colistin alone, had extensive tubular damage including tubular epithelial necrosis and vacuolation whereas ABGE-pretreated rats had significantly reduced tubular injury (Figure 2). Masson’s trichrome staining, the reliable method to present renal fibrosis, demonstrated that high signal was detected on colistin only group whereas this signal was attenuated by ABGE pretreatment. These results show that renal tissue injury after colistin administration could be prevented by ABG.

### Renal apoptosis

**Figure 3.**
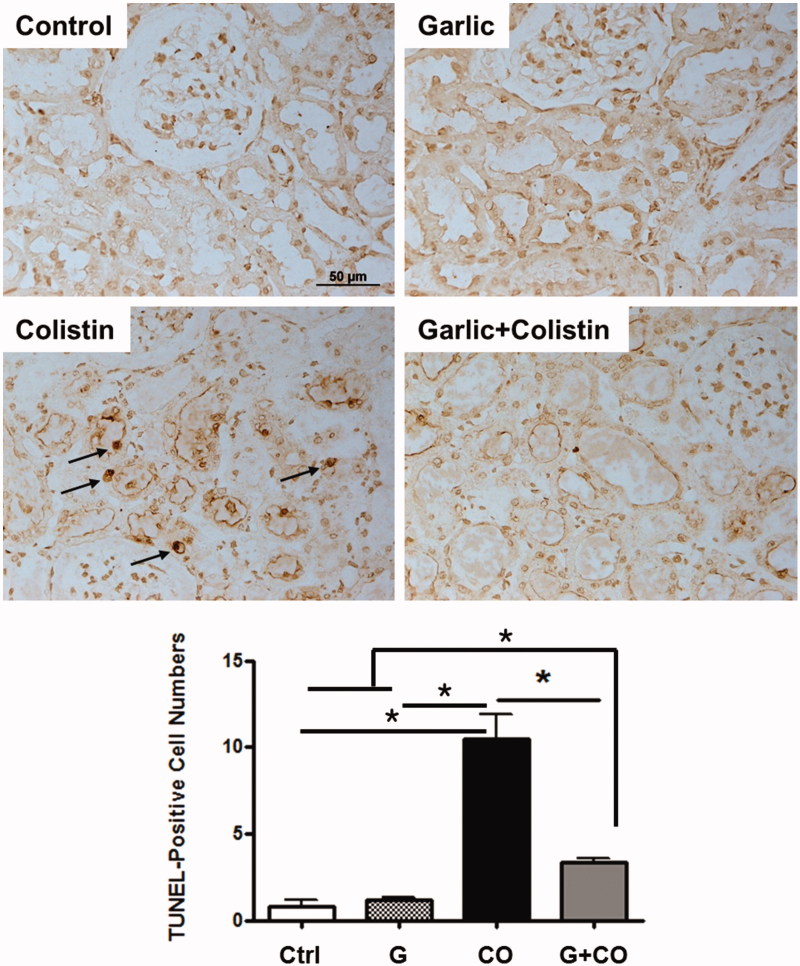
Effects of ABGE on tubular apoptotic renal injury by colistin. Colistin-induced apoptosis was illustrated by the TUNEL assay. TUNEL-positive cells were stained brown (arrow). To distinguish the nucleus, TUNEL-stained tissue sections were stained with hematoxylin (×400 original magnification). Rats were treated with sterile saline (Control), ABG (Garlic), colistin (Colistin), and ABGE plus colistin (Garlic + Colistin). TUNEL-positive cells were counted as described in ‘Material and Methods’. Figure shown is representative of four groups. Scale bar, 50 μm.

It is known that apoptosis-mediated tubular damage has been implicated in nephrotoxic drug-induced kidney injury [20–22]. We also examined the effects of garlic on colistin-induced tubular cell apoptosis by TUNEL staining (Figure 3). There was no change in the control and garlic groups. Clearly, colistin administration resulted in the increase of TUNEL positive cells, however, ABGE pretreatment significantly decreased the numbers of TUNEL-positive cells (Figure 3). This result presents that apoptosis plays a key role in renal toxicity of colistin injection.

### Oxidative stress

**Figure 4.**
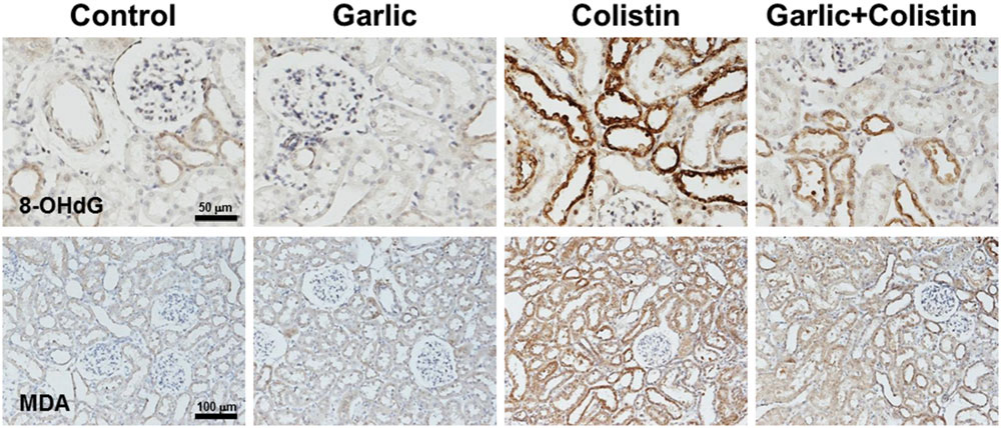
Reduced colistin-induced renal oxidative stress by ABGE. Immunohistochemical staining was performed with a specific antibody against 8-hydroxydeoxyguanosine (8-OHdG) and malondialdehyde (MDA). Rats were treated with sterile saline (Control), ABGE (Garlic), colistin (Colistin), and ABGE plus colistin (Garlic + Colistin). Figure shown are representative of the experiments. Scale bar, 50 μm and 100 μm for 8-OHdG and MDA, respectively.

Immunohistochemical staining of 8-OHdG, a reactive oxygen species (ROS)-induced DNA damage marker, and MDA, a representative marker of lipid peroxidation, were performed to investigate the effect of ABGE on colistin-induced oxidative stress. 8-OHdG-positive signals were highly detected in the nuclei of the damaged tubular epithelial cells. This signal was decreased by ABGE treatment (Figure 4). MDA-positive signals were also highly observed in the cytoplasm of the damaged tubular epithelial cells. This signal was also decreased by ABGE treatment as like 8-OHdG-positive signals (Figure 4).

### Renal macrophage

**Figure 5.**
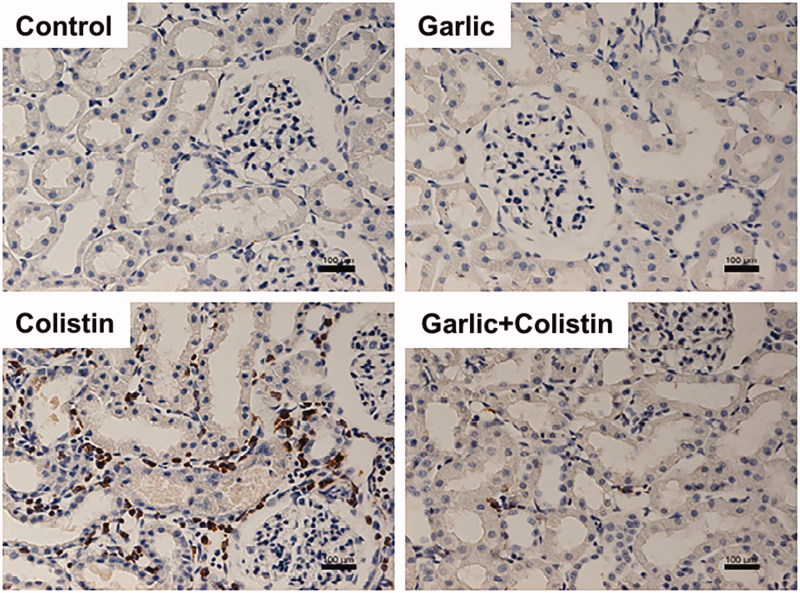
Reduced CD68 positive cells infiltration in the kidneys by ABGE after colistin injection. Immunohistochemical staining was performed with a specific antibody against CD68. Rats were treated with sterile saline (Control), ABGE (Garlic), colistin (Colistin), and ABGE plus colistin (Garlic + Colistin). Figure shown are representative of the experiments. Scale bar, 100 μm.

Garlic has anti-inflammatory effect. Macrophages, representative of inflammatory cells, highly express ED1 when activated, which is the rat homolog of human CD68, as a surface protein. Therefore, ED1-positive cells are considered as monocytes or macrophages in rats. Colistin increased infiltration of ED1-positive cells into the kidney. ABGE administration significantly decreased the infiltration of these cells (Figure 5).

### Renal inflammation

**Figure 6.**
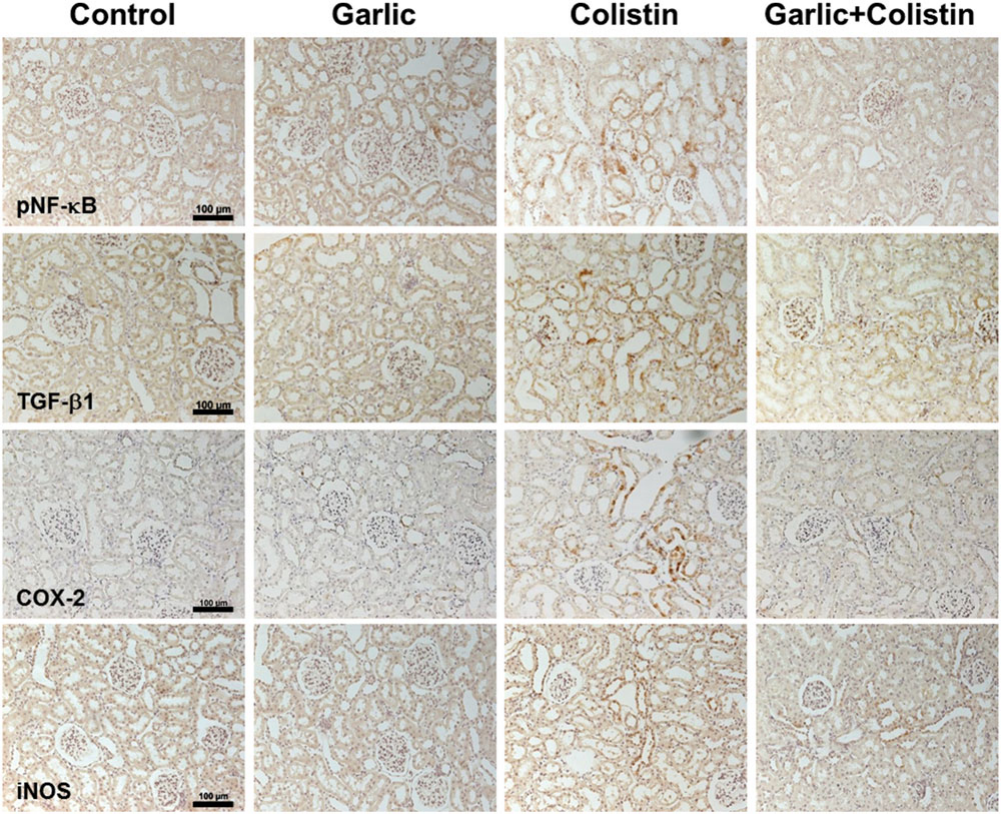
Decreased colistin-induced renal inflammations by ABGE. Immunohistochemical staining was performed with a specific antibody against NF-*k*B, TGF-*β*1, iNOS, and COX-2. Rats were treated with sterile saline (Control), ABGE (Garlic), colistin (Colistin), and ABGE plus colistin (Garlic + Colistin). Figure shown are representative of the experiments. Scale bar, 100 μm.

The NF-*κ*B signaling pathway is a major source of inflammation. We examined the activation of the NF-*κ*B signaling pathway and TGF-*β*1 expression as a target of the NF-*κ*B signaling. Marked induction of pNF-*κ*B and TGF-*β*1 protein was detected on colistin group (Figure 6.). Garlic reduced expression of these proteins. The major effector pathway of inflammatory macrophages is mediated by nitric oxide (NO) synthesized by inducible NO synthase (iNOS). iNOS expression was not detected in untreated rat kidneys by immunohistochemical staining. Notably, in kidneys of colistin rat, iNOS staining intensity was increased and localized mainly to the apical membranes of tubular epithelial cells. In contrast, ABGE treated rats exhibited less iNOS staining in tubular epithelial cells compared with colistin rats (Figure 6). Cyclooxygenase-2 (COX-2) is an inducible enzyme, becoming abundant in activated macrophages and other cells at sites of inflammation. Expression of COX-2 was also decreased in ABGE treated rats, compared to only colistin injected rats (Figure 6).

### Renal inflammatory factors and antioxidants measured by ELISA

**Figure 7.**
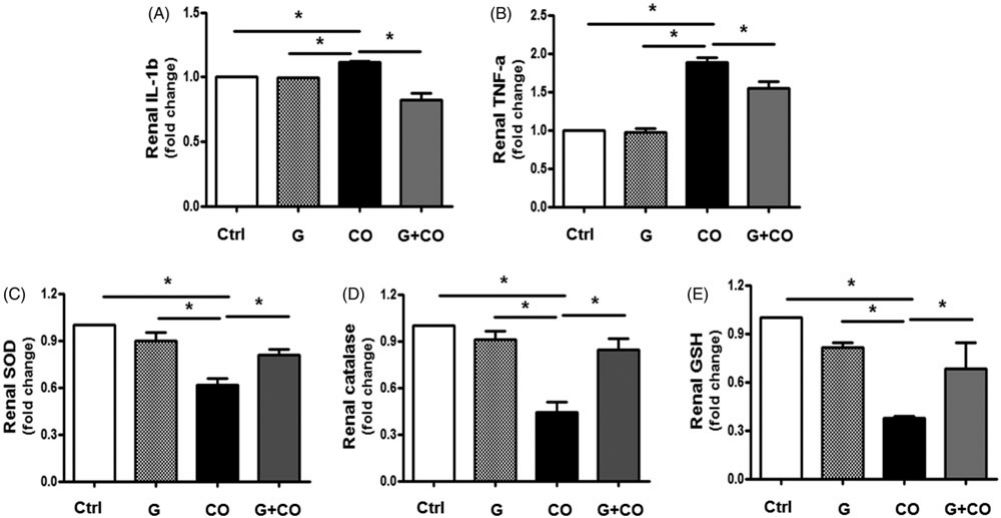
Anti-inflammatory and antioxidant effects of ABGE on the colistin-induced kidney injury. (A–E) renal IL-1*β*, TNF-*β*1, SOD, catalase, and GSH were measured by ELISA. The fold changes are calculated as the ratio of the final value in the other groups to the control group. Average value of each control individual is set as ‘1’. G: intragastric administration of saline solution with 1% ABGE (100 *µ*L per individual); CO: 10 mg/kg i.p. injection of colistin; G + CO: intragastric saline solution with 1% ABGE (100 *µ*L per individual) and 10 mg/kg i.p. injection of colistin for 6 consecutive days. Three independent experiments were performed, and the data are presented as mean ± SE (*p < 0.05).

Renal levels of IL-1*β* and TNF-*α*, quantified by ELISA, were increased by colistin administration compared to the levels in the control and garlic groups. Kidney in the rat given garlic and colistin showed the decreased these levels (Figure 7(A,B)). In addition, renal SOD, catalase, and GSH levels decreased significantly, while the levels increased with garlic treatment (Figure 7(C–E)).

## Discussions

Our study revealed that colistin-induced acute deterioration of renal function in rat kidney was associated with histopathological changes in renal tubule, increase in apoptotic cells, an increase of oxidative stress biomarkers such as 8-OHdG and MDA, and an increase of inflammation such as macrophage infiltration, NF-*k*B, TGF-*β*1, iNOS, and COX-2. Intragastric ABGE administration prevented renal dysfunction and its associated structural changes. These beneficial effects of ABGE might be originated from antioxidant and anti-inflammatory one represented by decrease of above markers.

Oxidative stress also has been presented to play a key role in the nephrotoxicity caused by other drugs such as gentamicin, vancomycin, and cisplatin. Mitochondrial ROS can damage many cellular macromolecules as like nucleic acid and proteins which can lead to cell death [27]. Practically, some studies in rats suggest that oxidative stress plays a significant role in colistin-induced nephrotoxicity. The administration of melatonin, *N*-acetylcysteine, and ascorbic acid, a series of antioxidant, had protective effect against colistin-induced nephrotoxicity [28–30]. Our study also proved that ABGE reduced colistin-induced kidney injury through inhibition of apoptotic cell death by suppressing the oxidative stress represented by reduction of 8-OHdG and MDA and restored antioxidant level such as renal SOD catalase and GSH.

Several studies have reported that garlic, garlic extracts, and constituents, in the fresh or aged forms, prevented renal dysfunction in gentamicin animal models, representative of nephrotoxic models [20,31–34]. Aged garlic extract prevented the GM-induced increase in the oxidative stress markers and decrease in antioxidant enzymes; manganese superoxide dismutase, glutathione peroxidase, and glutathione reductase activities [20]. The constituents of garlic, diallyl sulfide, and S-allylcysteine (SAC), also prevented gentamicin-induced nephrotoxicity by reducing oxidative stress [31,32]. However, there was no study for prevention of ABGE with antioxidant and anti-inflammatory effect to nephrotoxic drugs. We firstly demonstrated that ABGE prevented colistin-induced nephrotoxicity.

Garlic has been known as a food with antioxidant and anti-inflammatory effects [11–13]. It is known that ROS induce inflammation and antioxidants have anti-inflammatory effects [19]. ABGE is a type of fermented garlic used as food ingredient in Korea, Thailand, and Japan. It is made through aging procedures at high temperature and humidity during a long time. Because of aging processes, it has strong antioxidant activities, compared with fresh raw garlic [18,35]. Some reports demonstrated that aged garlic also has anti-inflammatory potency [11,19]. Inflammation is an important response to harmful stimuli and directly related with various diseases. Activated macrophages emit various pro-inflammatory molecules such as cytokines and NO which is produced by NOS [36]. iNOS is highly expressed in response to various inflammatory stimuli. Therefore, excessive NO production has been a target to control the inflammatory disease

Previous studies showed that garlic extracts modulated NO production by inhibiting inflammation-related transcription factors, such as NF-*κ*B or from induction of heme-oxygenase-1 (HO-1) [37–39]. Our study demonstrated that ABG administration was accompanied with reduction of CD68, iNOS, and COX-2 expression up-regulated by colistin injection. Intragastric ABGE administration also did significantly reduce colistin-induced NF-*κ*B activation although not increase HO-1 expression (data not shown) and renal IL-1*β* and TNF-*α* level. These results represented that the beneficial effect of ABGE on colistin-induced nephrotoxicity model in rats was derived from anti-inflammatory properties. This result is consistent with previous reports that ABGE had anti-inflammatory potency [11,19].

It is known that phytochemical compounds such as phenolic compounds, flavonoids, pyruvate and thiosulfate and the major organosulfur compounds such as SAC and S-allylmercaptocysteine are the main components of ABG which have antioxidant capacity although the overall composition of ABGE has not yet been analyzed [18,40]. It has not been known as a standard marker of ABGE. ABGE is produced by aging whole garlic at high temperature and high humidity. During the aging process, unstable compounds of fresh garlic including alliin are converted into stable compounds including SAC, the water-soluble compound with potent antioxidant effect. It is known that an increase in SAC during aging could be responsible for the stronger antioxidant activity of aged black garlic than that of garlic [41–43]. To measure SAC compounds or its metabolites in blood and/or urine might be used as a biomarker of ABGE [44].

In summary, to our knowledge, this is the first study demonstrating that ABGE had beneficial effects on protecting colistin-induced acute kidney injury. Our results suggest that administration of colistin might induce the production of ROS, which causes oxidative stress and protein oxidation, is mainly involved in the inflammation resulting in functional and structural renal damages. ABGE use with potent antioxidant effects might reduce the oxidative stress and protein oxidation indirectly leading to reduction of inflammation or have directly anti-inflammatory capacities. These results provide that ABGE might have therapeutic potentials in patients which colistin be used to prevent nephrotoxicity and get good clinical outcomes. Further study remains to be established.

## Ethics committee approval

Animal studies were conducted according to the guidelines for the care and handling of animals prepared by the Gyeongsang National University Institutional Animal Care & Ethics Committee (GNU-120615-R0024).

## References

[1] Zavascki AP, Goldani LZ, Li J, et al. Polymyxin B for the treatment of multidrug resistant pathogens: a critical review. J Antimicrob Chemother. 2007;60:1206–1215. 17878146 10.1093/jac/dkm357

[2] Michalopoulos AS, Tsiodras S, Rellos K, et al. Colistin treatment in patients with ICU-acquired infections caused by multidrug resistant gram negative bacteria: the renaissance of an old antibiotic. Clin Microbiol Infect. 2005;11:115–121. 15679485 10.1111/j.1469-0691.2004.01043.x

[3] Michalopoulos AS, Falagas ME Colistin and polymyxin B in critical care. Crit Care Clin. 2008;24:377–391. 18361952 10.1016/j.ccc.2007.12.003

[4] Falagas ME, Kasiakou SK Colistin: the revival of polymyxins for the management of multidrug-resistant gram-negative bacterial infections. Clin Infect Dis. 2005;40:1333–1341. 15825037 10.1086/429323

[5] Falagas ME, Fragoulis KN, Kasiakou SK, et al. Nephrotoxicity of intravenous colistin: a prospective evaluation. Int J Antimicrob Agents. 2005;26:504–507. 16280245 10.1016/j.ijantimicag.2005.09.004

[6] Hartzell JD, Neff R, Ake J, et al. Nephrotoxicity associated with intravenous colistin (colistimethate sodium) treatment at a tertiary care medical center. Clin Infect Dis. 2009;48:1724–1728. 19438394 10.1086/599225

[7] Kallel H, Hergafi L, Bahloul M, et al. Safety and efficacy of colistin compared with imipenem in the treatment of ventilator-associated pneumonia: a matched case-control study. Intensive Care Med. 2007;33:1162–1167. 17530220 10.1007/s00134-007-0675-2

[8] Ko HJ, Jeon MH, Choo EJ, et al. Early acute kidney injury is a risk factor that predicts mortality in patients treated with colistin. Nephron Clin Pract. 2011;117:c284–c288. 20847571 10.1159/000320746

[9] Garonzik SM, Li J, Thamlikitkul V, et al. Population pharmacokinetics of colistin methanesulfonate and formed colistin in critically ill patients from a multicenter study provide dosing suggestions for various categories of patients. Antimicrob Agents Chemother. 2011;55:3284–3294. 21555763 10.1128/AAC.01733-10 PMC3122440

[10] Wallace SJ, Li J, Nation RL, et al. Subacute toxicity of colistin methanesulfonate in rats: comparison of various intravenous dosage regimens. Antimicrob Agents Chemother. 2008;52:1159–1161. 18180359 10.1128/AAC.01101-07 PMC2258545

[11] Kim MJ, Yoo YC, Kim HJ, et al. Aged black garlic exerts anti-inflammatory effects by decreasing no and proinflammatory cytokine production with less cytoxicity in LPS-stimulated raw 264.7 macrophages and LPS-induced septicemia mice. J Med Food. 2014;17:1057–1063. 25238199 10.1089/jmf.2013.3043

[12] Shin JH, Ryu JH, Kang MJ, et al. Short-term heating reduces the anti-inflammatory effects of fresh raw garlic extracts on the LPS-induced production of NO and pro-inflammatory cytokines by downregulating allicin activity in RAW 264.7 macrophages. Food Chem Toxicol. 2013;58:545–551. 23583806 10.1016/j.fct.2013.04.002

[13] Chen S, Shen X, Cheng S, et al. Evaluation of garlic cultivars for polyphenolic content and antioxidant properties. PLoS One. 2013;8:e79730. 24232741 10.1371/journal.pone.0079730 PMC3827438

[14] Bordia T, Mohammed N, Thomson M, et al. An evaluation of garlic and onion as antithrombotic agents. Prostaglandins Leukot Essent Fatty Acids. 1996;54:183–186. 8860105 10.1016/s0952-3278(96)90014-9

[15] Cellini L, Di Campli E, Masulli M, et al Inhibition of *Helicobacter pylori* by garlic extract (*Allium sativum*). FEMS Immunol Med Microbiol. 1996;13:273–277. 8739190 10.1111/j.1574-695X.1996.tb00251.x

[16] Alpers DH Garlic and its potential for prevention of colorectal cancer and other conditions. Curr Opin Gastroenterol. 2009;25:116–121. 19528879 10.1097/MOG.0b013e32831ef221

[17] Shin JH, Lee CW, Oh SJ, et al. Hepatoprotective effect of aged black garlic extract in rodents. Toxicol Res. 2014;30:49–54. 24795800 10.5487/TR.2014.30.1.049 PMC4007044

[18] Lee YM, Gweon OC, Seo YJ, et al. Antioxidant effect of garlic and aged black garlic in animal model of type 2 diabetes mellitus. Nutr Res Pract. 2009;3:156–161. 20016716 10.4162/nrp.2009.3.2.156 PMC2788179

[19] Jeong YY, Ryu JH, Shin JH, et al. Comparison of anti-oxidant and anti-inflammatory effects between fresh and aged black garlic extracts. Molecules. 2016;21:430. 27043510 10.3390/molecules21040430 PMC6274159

[20] Maldonado PD, Barrera D, Medina-Campos ON, et al. Aged garlic extract attenuates gentamicin induced renal damage and oxidative stress in rats. Life Sci. 2003;73:2543–2556. 12967679 10.1016/s0024-3205(03)00609-x

[21] Razo-Rodríguez AC, Chirino YI, Sánchez-González DJ, et al. Garlic powder ameliorates cisplatin-induced nephrotoxicity and oxidative stress. J Med Food. 2008;11:582–586. 18800910 10.1089/jmf.2008.0033

[22] Sener G, Sakarcan A, Yegen BC Role of garlic in the prevention of ischemia-reperfusion injury. Mol Nutr Food Res. 2007;51:1345–1352. 17966137 10.1002/mnfr.200700078

[23] Nasiri A, Ziamajidi N, Abbasalipourkabir R, et al. Beneficial effect of aqueous garlic extract on inflammation and oxidative stress status in the kidneys of type 1 diabetic rats. Indian J Clin Biochem. 2017;32:329–336. 28811693 10.1007/s12291-016-0621-6 PMC5539014

[24] Ziamajidi N, Nasiri A, Abbasalipourkabir R, et al. Effects of garlic extract on TNF-α expression and oxidative stress status in the kidneys of rats with STZ + nicotinamide-induced diabetes. Pharm Biol. 2017;55:526–531. 27937047 10.1080/13880209.2016.1255978 PMC6130558

[25] Ziamajidi N, Behrouj H, Abbasalipourkabir R, et al. Ameliorative effects of *Allium sativum* extract on iNOS gene expression and NO production in liver of streptozotocin + nicotinamide-induced diabetic rats. Ind J Clin Biochem. 2018;33:147–153. 10.1007/s12291-017-0656-3 PMC5891448 29651204

[26] Kim JH, Lee SS, Jung MH, et al. N-acetylcysteine attenuates glycerol-induced acute kidney injury by regulating MAPKs and Bcl-2 family proteins. Nephrol Dial Transplant. 2010;25:1435–1443. 20037173 10.1093/ndt/gfp659

[27] Lopez-Novoa JM, Quiros Y, Vicente L, et al. New insights into the mechanism of aminoglycoside nephrotoxicity: an integrative point of view. Kidney Int. 2011;79:33–45. 20861826 10.1038/ki.2010.337

[28] Ozyilmaz E, Ebinc FA, Derici U, et al. Could nephrotoxicity due to colistin be ameliorated with the use of N-acetylcysteine? Intensive Care Med. 2011;37:141–146. 20845026 10.1007/s00134-010-2038-7

[29] Yousef JM, Chen G, Hill PA, et al. Melatonin attenuates colistin-induced nephrotoxicity in rats. Antimicrob Agents Chemother. 2011;55:4044–4049. 21709095 10.1128/AAC.00328-11 PMC3165279

[30] Yousef JM, Chen G, Hill PA, et al. Ascorbic acid protects against the nephrotoxicity and apoptosis caused by colistin and affects its pharmacokinetics. J Antimicrob Chemother. 2012;67:452–459. 22127588 10.1093/jac/dkr483 PMC3254197

[31] Pedraza-Chaverrí J, Maldonado PD, Barrera D, et al. Protective effect of diallyl sulfide on oxidative stress and nephrotoxicity induced by gentamicin in rats. Mol Cell Biochem. 2003;254:125–130. 14674690 10.1023/a:1027372102135

[32] Maldonado PD, Barrera D, Rivero I, et al. Antioxidant S-allylcysteine prevents gentamicin-induced oxidative stress and renal damage. Free Radic Biol Med. 2003;35:317–324. 12885594 10.1016/s0891-5849(03)00312-5

[33] Pedraza-Chaverrí J, Maldonado PD, Medina-Campos ON, et al. Garlic ameliorates gentamicin nephrotoxicity: relation to antioxidant enzymes. Free Radic Biol Med. 2000;29:602–611. 11033412 10.1016/s0891-5849(00)00354-3

[34] Nasri H, Nematbakhsh M, Rafieian-Kopaei M Ethanolic extract of garlic for attenuation of gentamicin-induced nephrotoxicity in Wistar rats. Iran J Kidney Dis 2013;7:376–382. 24072150

[35] Kim MH, Kim MJ, Lee JH, et al. Hepatoprotective effect of aged black garlic on chronic alcohol-induced liver injury in rats. J Med Food. 2011;14:732–738. 21663494 10.1089/jmf.2010.1454

[36] Pan CH, Kim ES, Jung SH, et al. Tectorigenin inhibits IFN-gamma/LPS-induced inflammatory responses in murine macrophage RAW 264.7 cells. Arch Pharm Res. 2008;31:1447–1456. 19023541 10.1007/s12272-001-2129-7

[37] Ban JO, Oh JH, Kim TM, et al Anti-inflammatory and arthritic effects of thiacremonone, a novel sulfur compound isolated from garlic via inhibition of NF-kappaB. Arthritis Res Ther. 2009;11:R145 19788760 10.1186/ar2819 PMC2787296

[38] Park HJ, Jeon BT, Kim HC, et al. Aged red garlic extract reduces lipopolysaccharide-induced nitric oxide production in RAW 264.7 macrophages and acute pulmonary inflammation through haeme oxygenase-1 induction. Acta Physiol. 2012;205:61–70. 10.1111/j.1748-1716.2012.02425.x 22353229

[39] Ryu JH, Park HJ, Jeong YY, et al. Aged red garlic extract suppresses nitric oxide production in lipopolysaccharide-treated RAW 264.7 macrophages through inhibition of NF-κB. J Med Food. 2015;18:439–445. 25584924 10.1089/jmf.2014.3214

[40] Lee SJ, Shin JH, Kang MJ, et al. Antioxidants activity of aged red garlic. J Life Sci. 2010;20:775–781.

[41] Jang EK, Seo JH, Lee SP Physiological activity and antioxidative effects of aged black garlic (*Allium sativum* L.) extract. Korean Soc Food Sci Technol. 2008; 40:443–448.

[42] Kang MJ, Lee SJ, Shin JH, et al. Effect of garlic with different processing on lipid metabolism in 1% cholesterol fed rats. J Korean Soc Food Sci Nutr.2008;37:162–169.

[43] Corzo-Martinez M, Corso N, Villamiel M Biological properties of onions and garlic. Trends Food Sci Technol. 2007;18:609–625.

[44] Amano H, Kazamori D, Itoh K Pharmacokinetics of S-allyl-l-cysteine in rats is characterized by high oral absorption and extensive renal reabsorption. J Nutr. 2016;146:456S–459S. 26764325 10.3945/jn.114.201749

